# Enhanced sympathetic activity and cardiac sympathetic afferent reflex in rats with heart failure induced by adriamycin

**DOI:** 10.7555/JBR.26.20120035

**Published:** 2012-10-19

**Authors:** Shujuan Zhang, Feng Zhang, Haijian Sun, Yebo Zhou, Ying Han

**Affiliations:** Department of Physiology, Nanjing Medical University, Nanjing, Jiangsu 210029, China

**Keywords:** heart failure, adriamycin, sympathetic activity, angiotensin II, paraventricular nucleus

## Abstract

Our previous studies have shown that the cardiac sympathetic afferent reflex is enhanced in rats with chronic heart failure (CHF) induced by coronary artery ligation and contributes to the over-excitation of sympathetic activity. We sought to determine whether sympathetic activity and cardiac sympathetic afferent reflex were enhanced in adriamycin-induced CHF and whether angiotensin II (Ang II) in the paraventricular nucleus (PVN) was involved in enhancing sympathetic activity and cardiac sympathetic afferent reflex. Heart failure was induced by intraperitoneal injection of adriamycin for six times during 2 weeks (15 mg/kg). Six weeks after the first injection, the rats underwent anesthesia with urethane and α-chloralose. After vagotomy and baroreceptor denervation, cardiac sympathetic afferent reflex was evaluated by renal sympathetic nerve activity and mean arterial pressure (MAP) response to epicardial application of capsaicin (1.0 nmol). The response of MAP to ganglionic blockade with hexamethonium in conscious rats was performed to evaluate sympathetic activity. The renal sympathetic nerve activity and cardiac sympathetic afferent reflex were enhanced in adriamycin rats and the maximum depressor response of MAP induced by hexamethonium was significantly greater in adriamycin rats than that in control rats. Bilateral PVN microinjection of angiotensin II (Ang II) caused larger responses of the cardiac sympathetic afferent reflex, baseline renal sympathetic nerve activity and MAP in adriamycin rats than control rats. These results indicated that both sympathetic activity and cardiac sympathetic afferent reflex were enhanced and Ang II in the PVN was involved in the enhanced sympathetic activity and cardiac sympathetic afferent reflex in rats with adriamycin-induced heart failure.

## INTRODUCTION

It is well known that sympathetic activity is over-excited in chronic heart failure (CHF) induced by coronary artery ligation[1],[2]. Persistent sympathoexcitatory state contributes to hemodynamic deterioration and the pathogenesis and progression of organ damage in the disease[3],[4]. Cardiac sympathetic afferent reflex (CSAR) is known as a positive-feedback, sympathoexcitatory cardiovascular reflex. It is induced by stimulation of cardiac sympathetic afferents with endogenous chemicals such as adenosine, bradykinin and hydrogen peroxide released from the myocardium during myocardial ischemia. This excitatory reflex results in sympathetic activation[5]. The CSAR can be elicited by direct electrical stimulation of cardiac sympathetic afferent nerves or epicardial application of exogenous chemicals such as bradykinin or capsaisin and contribute to the increased sympathetic activity and blood pressure[6]. Our previous studies have shown that enhanced CSAR contributes to excessive sympathetic activity which is involved in the aggravation of CHF induced by coronary artery ligation[7],[8]. Inhibition of enhanced CSAR and sympathetic tone may be beneficial for CHF.

It is known that myocardial damage leads to CHF. Adriamycin (ADR) is a potent and broad-spectrum antineoplastic agent that plays a major role in cancer chemotherapy. But unfortunately, ADR is particularly toxic to the heart tissue, which causes diffuse myocardial damage[9]–[12] and induces CHF[13],[14] by increased oxidative stress and apoptosis. However, whether sympathetic activity and the CSAR are also enhanced in cardiomyopathy-induced CHF is still unknown. Our previous studies have shown that the paraventricular nucleus (PVN) is an important component of the central neurocircuitry of the CSAR[15]. Angiotensin II (Ang II) in the PVN augments the CSAR which is prevented by pretreatment with AT_1_ receptor antagonist losartan in rats with CHF caused by coronary artery ligation[1],[16].

The present study was designed to determine whether the CSAR and sympathetic activity were enhanced in ADR-induced heart failure rats and whether Ang II in the PVN was involved in the enhanced sympathetic activity and CSAR in ADR rats.

## MATERIALS AND METHODS

### Drugs

Capsaicins, Ang II, and hexamethonium were obtained from Sigma (St Louis, MO, USA). ADR was obtained from Zhejiang Hisun Pharmaceutical Co., Ltd. All drugs were dissolved in normal saline.

### Animals

Experiments were carried out in male Sprague-Dawley rats which were maintained in cages in a temperature-controlled room regulated on a 12 h-12 h light-dark cycle with free access to standard rat chow and tap water. The experimental procedures were approved by the Experimental Animal Care and Use Committee of Nanjing Medical University and complied with the Guide for the Care and Use of Laboratory Animals (NIH publication No. 85-23, revised 1996).

### Model of ADR-induced heart failure

ADR-induced heart failure rat model was established as previously described[17],[18]. ADR (doxorubicin hydrochloride) was administered intraperitoneally in six equal injections (each containing ADR 2.5 mg/kg, body weight) to rats weighing between 180 and 220 g over a period of 2 weeks for a total dose of 15 mg/kg body weight[19]–[21]. After final injection, rats were observed for 4 weeks for their general appearance, behavior, and mortality. The control rats were handled in the same procedures except using saline instead of ADR.

### Ganglionic blockade with hexamethonium hydrochloride

The response of mean arterial pressure (MAP) to ganglionic blockade with hexamethonium hydrochloride was used to evaluate sympathetic activity in conscious rats[22],[23]. The right carotid artery and jugular vein were cannulated for recording of MAP and administration of chemicals in rats under chloral hydrate (300 mg/kg). At least 3 h after recovery from anesthesia[22], the baseline MAP was recorded and then 30 mg/kg of hexamethonium hydrochloride was injected into the conscious rats via the jugular vein. The maximal decrease in MAP was considered as an index to evaluate sympathetic activity[24],[25].

### Hemodynamic measurements

Six weeks after the first intraperitoneal injection of ADR or saline, cardiac functions of rats were assessed hemodynamically. Each rat was anesthetized with intraperitoneal injection of urethane (800 mg/kg) and α-chloralose (40 mg/kg). Adequate depth of anesthesia was maintained by supplemental doses of anesthesia during experiment. The trachea was cannulated for mechanical ventilation using a rodent ventilator (Model 683, Harvard Apparatus, South Natick, MA, USA). A catheter connected with a pressure transducer (MLT0380, ADInstruments, NSW, Australia) was placed into the left ventricle (LV) for measuring the LV peak systolic pressure (LVSP), LV end-diastolic pressure (LVEDP) and the maximum of the first differentiation of left ventricular pressure (+LVdp/dt_max_). The criteria for CHF were an elevated LVEDP (>13 mmHg), and a 40% decrease in +LVdp/dt_max_[1].

**Table 1:** Anatomical and hemodynamic data in the control and experimental rats

Variables	Control	Adriamycin
Body weight (g)	421.40±2.70	375.90±4.10*
Heart weight (g)	001.30±0.01	000.90±0.02*
Heart weight/Body weight (g/kg)	003.10±0.03	002.40±0.05*
Mean arterial pressure (mm Hg)	094.80±1.50	*93.40±1.50
Heart rate (beats/min)	360.40±5.70	384.90±8.000
LV peak systolic pressure (mm Hg)	138.70±2.30	100.70±1.40*
LV end-diastolic pressure (mm Hg)	000.30±0.60	012.40±0.40*
+LVdP/dt_max_ (mm Hg/sec)	3404.30±81.10	2376.40±53.20*
Mortality (%)	0	32

LV: left ventricle; +LVdP/dt_max_: the maximum of the first differentiation of left ventricular pressure. Data are expressed as mean±SE. *P < 0.05 compared with control rats.

### Vagotomy and baroreceptor denervation

After hemodynamic measurement, the catheter connected with the pressure transducer was placed in the carotid artery for continuous recording of MAP and heart rate (HR). Vagotomy and baroreceptor denervation were carried out to minimize confounding effect of the baroreflex on sympathetic activity and blood pressure as described in our previous report[26]. The bilateral vagi and carotid sinus nerves were identified in the neck, tied and sectioned. The common carotid arteries and carotid bifurcation were painted with 10% phenol solution to destroy any remaining nerve fibers in this area. The effect of baroreceptor denervation was identified by criterion of the HR change less than 5 beats/min after intravenous injection of phenylephrine (20 µg/kg) that induced an increase in MAP between 25 and 40 mm Hg.

### Recording of renal sympathetic nerve activity (RSNA)

RSNA was recorded as described in our previous reports[7],[27]. The left renal sympathetic nerve was isolated through a retroperitoneal incision and cut distally to eliminate the renal afferent activity under an operating microscope. The nerve was placed on a pair of silver recording electrodes and immersed in mineral oil. The nerve signals were amplified with an AC/DC differential amplifier (Model 3000, A-M System Inc.) with a low frequency cutoff of 60 Hz and a high-frequency cutoff of 3 kHz. The amplified and filtered signals were integrated at time constant of 100 ms and background noise was determined at the end of each experiment. RSNA, MAP and HR were simultaneously recorded on a PowerLab data acquisition system (8SP, ADInstruments, Sydney, Australia). RSNA was expressed as the percent change from control after each intervention.

### Evaluation of CSAR

Left lateral thoracotomy was performed and the pericardium was removed. CSAR was elicited by epicardial application of a piece of filter paper (3×3 mm) containing capsaicin (1.0 nmol in 2.0 µL) on the anterior wall of the left ventricle for 1 min. The epicardium was then rinsed three times with 10 mL of warm normal saline (38°C). CSAR was evaluated by the RSNA and MAP responses to the epicardial application of capsaicin[27].

### PVN microinjection

Rats were placed in a stereotaxic frame (Stoelting, Chicago, IL, USA). Stereotaxic coordinates for the PVN were determined according to the Paxinos and Watson rat atlas, which were 1.8 mm caudal from the bregma, 0.4 mm lateral to the midline and 7.9 mm ventral to the dorsal surface. The microinjection volume was 50 nL for each PVN and completed within 1 min. At the end of experiment, 50 nL of Evans blue (2%) was injected into each microinjection site for histological identification. Rats with microinjection sites outside the PVN were excluded from data analysis.

### Protocol

All measurements were carried out at the end of the sixth week after the first intraperitoneal injection of ADR or saline in rats. Firstly, hemodynamic data were collected in ADR and control rats. Secondly, the responses of MAP to ganglionic blockade with hexamethonium hydrochloride in one group of conscious ADR rats and one group of conscious control rats were respectively determined (*n* = 6 for each group). Thirdly, the CSAR and effects of Ang II in the PVN on RSNA, MAP and CSAR in anaesthetic control and ADR rats were determined. Both control and ADR rats were randomly divided into 2 groups (*n* = 6 for each group), which were respectively subjected to the PVN microinjection of saline or Ang II (0.3 nmol). The CSAR was determined before and 3 min after PVN microinjection.

### Statistical analysis

Comparisons between two observations in the same animal were assessed using Student's paired t-test. One-way ANOVA was used when multiple comparisons were made followed with the Bonferroni test for post hoc analysis. All data were expressed as mean± SE. *P* < 0.05 was considered statistically significant.

## RESULTS

### General characteristics

During the six weeks after the first intraperitoneal injection of ADR, a total of 31 ADR rats died and were excluded from analysis. The mortality for ADR rats was 32% and for control rats 0%. The ratios of body weight, heart weight and heart-to-body weight in the ADR rats decreased compared with those in the control rats (***Table 1***).

### Hemodynamics assessments

LVSP and + LVdP/dt_max_ were significantly lower and LVEDP was dramatically higher in the ADR rats compared with those in the control rats. There was no significant difference in MAP between the ADR and control rats (***Table 1***). These hemodynamic and anatomical data indicated the presence of myocardial damage and suggested decreased cardiac contractile function in ADR-induced heart failure rats.

**Fig. 1:**
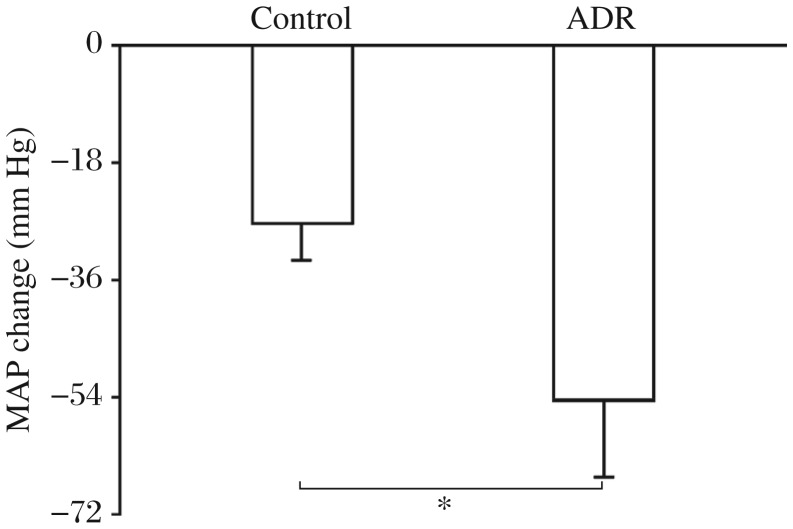
The response of mean arterial pressure (MAP) to ganglionic blockade of hexamethonium for evaluating sympathetic activity in the control and adriamycin (ADR) rats. The Values were expressed as mean± SE. *P < 0.05, compared with the control rats, *n* = 6 for each group.

### The response of MAP to ganglionic blockade with hexamethonium

The maximum depressor response of MAP induced by ganglionic blockade with intravenous injection of hexamethonium hydrochloride was significantly greater in the ADR rats than that in the control rats. The result suggested that the sympathetic activity was increased in the ADR rats compared with the control rats (***Fig. 1***).

### The CSAR

**Fig. 2:**
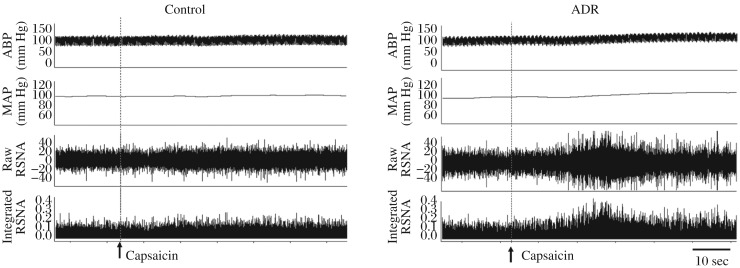
Representative tracings showing that cardiac sympathetic afferent reflex (CSAR) induced by epicardial application of capsaicin in the control and ADR rats. The CSAR was enhanced in the ADR rats. RSNA: renal sympathetic nerve activity; MAP: mean arterial pressure; ADR: adriamycin.

**Fig. 3:**
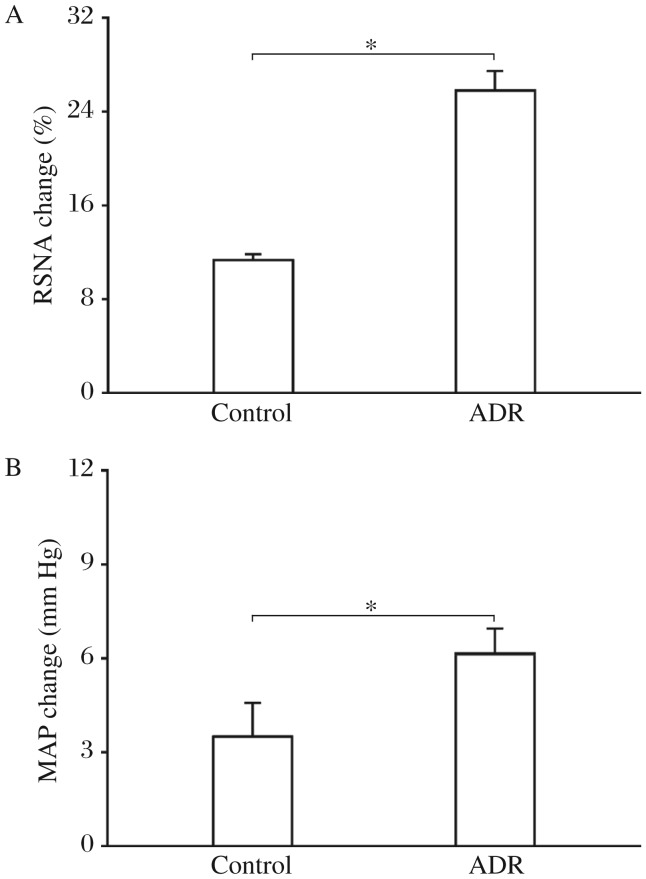
The CSAR in the control and ADR rats. The CSAR was evaluated by the RSNA and MAP responses to epicardial application of capsaicin (1 nmol). A: RSNA change. B: MAP change. The values were expressed as mean±SE. *P < 0.05, *n* = 6 for each group. CSAR: cardiac sympathetic afferent reflex; RSNA: renal sympathetic nerve activity; MAP: mean arterial pressure; ADR: adriamycin.

The representative recordings showed that the CSAR evoked by epicardial application of capsaicin was enhanced in the ADR rat compared with the control rat (***Fig. 2***). The CSAR, which was evaluated by the RSNA and MAP responses to epicardial application of capsaicin, was significantly enhanced in rats with ADR-induced heart failure (***Fig. 3***).

**Fig. 4:**
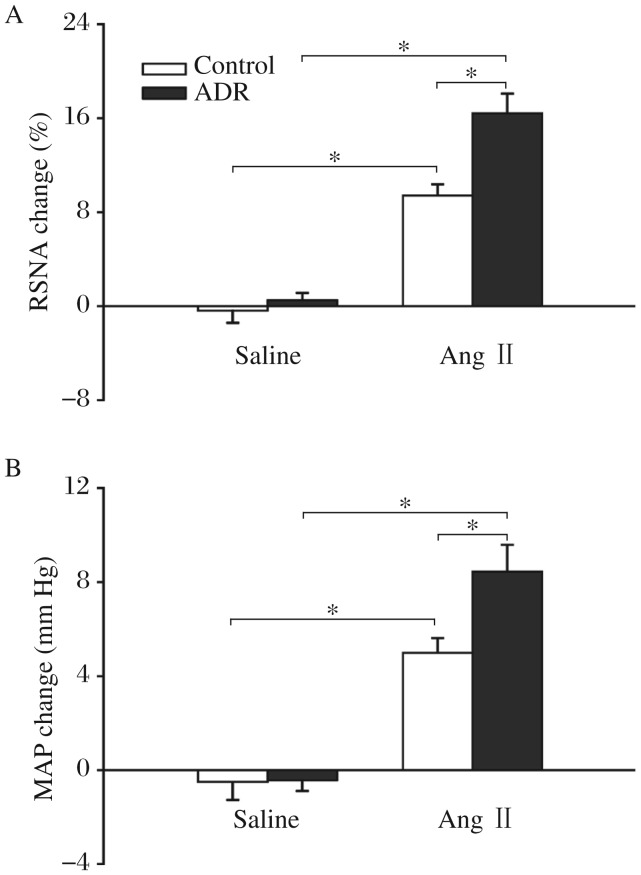
Effects of the paraventricular nucleus (PVN) microinjection of saline and angiotensin II (Ang II) on the baseline RSNA and MAP in the control and ADR rats. A: RSNA change. B: MAP change. The Values were expressed as mean±SE. *P < 0.05, *n* = 6 for each group. RSNA: renal sympathetic nerve activity; MAP: mean arterial pressure; ADR: adriamycin.

### Effects of Ang II in the PVN on the baseline RSNA and MAP and CSAR

**Fig. 5:**
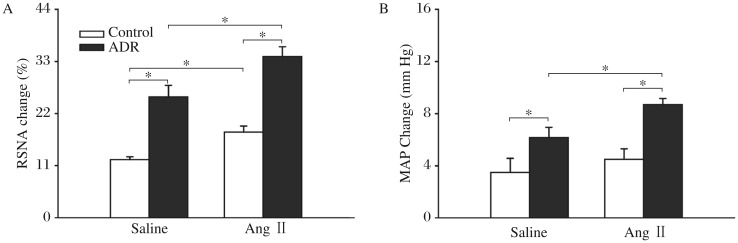
Effects of the PVN microinjection of saline and Ang II on the CSAR in the control and ADR rats. CSAR was evaluated by the RSNA and MAP responses to epicardial application of capsaicin (1 nmoL). A: RSNA change. B: MAP change. The values were expressed as mean±SE. *P < 0.05, *n* = 6 for each group. CSAR: cardiac sympathetic afferent reflex; RSNA: renal sympathetic nerve activity; MAP: mean arterial pressure; ADR: adriamycin; PVN: paraventricular nucleus.

Microinjection of Ang II into the PVN increased baseline RSNA and MAP in both ADR rats and control rats compared with saline. However, the RSNA and MAP changes caused by Ang II were much greater in the ADR rats than those in the control rats (***Fig. 4***). Microinjection of Ang II into the PVN enhanced the CSAR significantly in the ADR rats compared with saline. The CSAR response caused by Ang II was significantly greater in the ADR rats than that in the control rats (***Fig. 5***).

## DISCUSSION

Our previous studies have shown that CSAR is enhanced in rats with coronary artery ligation-induced CHF and contributes to the sympathetic overactivity[1],[2]. The excessive increased sympathetic activity results in hemodynamic deterioration. The pathogenesis and progression of organ damage in this disease were related to the prognosis for survival in CHF. Inhibition of the enhanced CSAR and sympathetic activity may be beneficial for coronary artery ligation-induced CHF.

ADR is one of the most effective anti-tumor drugs, but its clinical application is limited by risks of causing diffuse myocardial damage and developing heart failure. However, whether sympathetic activity and the CSAR are also enhanced in this myocardial damage induced CHF is still unknown. The treatment of ADR has many acute and chronic side effects. One of the major chronic side effects of ADR is particularly toxic to the heart tissue, which may be exerted via increased oxidative stress and apoptosis, causing diffuse myocardial damage[9]–[12] and ultimately CHF[13],[14]. ADR toxicity is characterized by hypotension, tachycardia and various arrhythmias[28] as well as reduced food intake and inhibition of protein synthesis, which result in body weight loss[29]. Six weeks after the first intraperitoneal injection of ADR, the ratios of body weight, heart weight and heart-to-body weight decreased, and the LVEDP increased while +LVdP/dt_max_ and LVSP decreased in the ADR rats compared with the control rats. These hemodynamic and anatomical data indicate the presence of myocardial damage and suggest decreased cardiac contractile function in the ADR rats. In addition, intraperitoneal injection of ADR successfully induced heart failure.

In the present study, the maximum depressor response of MAP induced by ganglionic blockade with hexamethonium was significantly greater in the ADR rats than that in the control rats, which suggested that sympathetic activity was increased in the ADR rats. The CSAR induced by epicardial application of capsaicin was enhanced in the ADR rats compared with the control rats. The enhanced CSAR may contribute to the over-excitation of sympathetic activity in rats with ADR-induced heart failure.

The PVN plays a major role in the integration of the sympathetic outflow and cardiovascular activity via projections to the intermediolateral column of the spinal cord and the rostral ventrolateral medulla[30], and is an important component of the central neurocircuitry of the CSAR[15]. Our previous studies have shown that microinjection of Ang II into the PVN augments the CSAR, which was prevented by PVN pretreatment with AT_1_ receptor antagonist losartan in normal rats[31] and in rats with coronary artery ligation-induced CHF[1],[16],[32]. The enhanced CSAR in rats with CHF induced by coronary artery ligation was normalized by microinjection of the AT_1_ receptor antagonist losartan, AT_1_ receptor mRNA antisense or the angiotensin-converting enzyme inhibitor captopril into the PVN[32],[33]. These results suggest that Ang II and AT_1_ receptors play important roles in the PVN in the enhancement of CSAR and sympathetic activity in coronary artery ligation-induced CHF. In the present study, microinjection of Ang II into the PVN increased baseline RSNA and MAP in both the ADR rats and control rats, and enhanced the CSAR in ADR rats compared with saline. The RSNA, MAP increasing and CSAR enhancing responses to Ang II were much greater in the ADR rats than those in the control rats. These results indicated that exogenous Ang II in the PVN plays an important role in modulating the enhanced CSAR and sympathetic activity in ADR rats. Combined with our previous reports, we speculated that a potential mechanism responsible for intensified responses to Ang II may be the up-regulated AT_1_ receptors in the PVN in the ADR rats compared with the control rats. This would be investigated in future studies.

In conclusion, the CSAR and sympathetic activity are enhanced in ADR-induced heart failure rats and Ang II in the PVN is involved in the enhanced CSAR and sympathetic activity in the ADR rats.
